# Challenges and opportunities for use of long-lasting insecticidal nets to prevent malaria during overnight travel in Uganda: a qualitative study

**DOI:** 10.1186/s12936-021-03811-1

**Published:** 2021-06-26

**Authors:** Deborah Ekusai-Sebatta, Emmanuel Arinaitwe, Arthur Mpimbaza, Joaniter I. Nankabirwa, Chris Drakeley, Philip J. Rosenthal, Sarah G. Staedke, Herbert Muyinda

**Affiliations:** 1 Infectious Diseases Research Collaboration, Kampala, Uganda; 2 London School of Hygiene and Tropical Medicine, London, UK; 3 Child Health & Development Centre, Makerere University College of Health Sciences, Kampala, Uganda; 4 Department of Medicine, Makerere University, Kampala, Uganda; 5 University of California, San Francisco, California USA

**Keywords:** Use of LLINs, Overnight travel, Malaria prevention

## Abstract

**Background:**

Travel is a well-recognized risk factor for malaria. Within sub-Saharan Africa, travellers from areas of lower to higher transmission intensity are potentially at high risk of malaria. Long-lasting insecticidal nets (LLINs) are the primary tool for prevention of malaria, and their widespread use has contributed to substantial reductions in malaria burden. However, travellers often fail to use LLINs. To further explore the challenges and opportunities of using LLINs, travellers were interviewed in Uganda.

**Methods:**

In August and September 2019, 20 participants attending outpatient clinics at Naguru General Hospital in Kampala with a history of travel out of Kampala within the previous 60 days were purposively selected. Data were collected through in-depth interviews and analysed thematically using NVivo 12.

**Results:**

Of the 20 participants, 13 were male. Thirteen of the 20 participants tested positive for malaria by microscopy, and 5 reported using of LLINs during travel. The main reasons for travel were to attend social events (weddings, funerals, overnight prayers) and for work. travellers who attended social events reported using LLINs less commonly than those who travelled for work. Challenges to using LLINs during travel included: (1) limited access to LLINs; (2) challenges in planning ahead of travel; (3) lack of space or ability to hang LLINs while travelling; (4) impression that LLINs in lodging places were unhygienic; (5) cultural beliefs discouraging use of LLINs during social events; (6) participation in overnight ceremonies; and (7) doubts about efficacy of LLINs. Positive factors influencing use of LLINs during travel included knowledge regarding malaria prevention and good affordability and availability of LLINs.

**Conclusions:**

Despite good traveller knowledge regarding malaria control measures, use of LLINs was limited. Use of LLINs in the prevention of malaria among travellers from low to high transmission settings needs to be prioritized. This calls for increased behaviour change oriented communication to improve traveller preparedness and consideration of use of repellents in situations where LLINs may not be feasible. The Uganda Ministry of Health and Malaria Control Division should use educational messages to increase awareness about the risks of getting malaria during overnight travel through the media. Truck drivers should be sensitized through their companies to use the available space at the back of the trucks for hanging nets and consider using pop-up nets.

**Supplementary Information:**

The online version contains supplementary material available at 10.1186/s12936-021-03811-1.

## Background

Travel is a well-documented risk factor for malaria transmission that if not addressed can undermine malaria prevention efforts [1, 2]. While substantial progress has been made in the control of malaria in Africa, much remains to be done to achieve prevention among travellers [3, 4]. Malaria transmission has become relatively low in many urban areas [5–7], resulting in urban residents having relatively low immunity, and so being at particular risk of malaria infection when they travel to rural areas [8]. There are proven malaria prevention measures, including use of long-lasting insecticidal nets (LLINs), that are recommended for travellers from low to highly malaria-endemic areas [9]. However, residents of malaria-endemic countries in sub-Saharan Africa often do not use LLINs while travelling away from home, which presents a challenge to malaria control and elimination efforts [10–12].

Uganda is highly endemic for malaria, with an estimated 90% of the population at risk [13]. However, malaria prevention measures that have been scaled up across the country over the last two decades have been associated with only modest reductions in the malaria burden [14]. While malaria transmission has become relatively low in many urban areas in Uganda, including Kampala [5–7], many malaria cases are diagnosed at public health facilities in these areas [15]. Many of these cases may be due to travel outside of urban areas and non-use of LLINs during overnight travel may contribute to increased risk among travellers [16]. Usage has been reported to be low in residents who travel from areas of relatively low to high malaria risk [1, 13]. A study conducted in Uganda showed that Kampala residents who travelled overnight out of the city were at substantially higher risk of malaria, with women less likely to use LLINs during travel than men [8]. However, information on use of malaria preventive measures in travellers is limited [17].

To explore further whether travellers use LLINs and other personal protection measures to prevent malaria, and more importantly, why they do not, a qualitative study was conducted.Local perceptions and attitudes were analysed regarding use of LLINs and other malaria prevention practices among individuals travelling from Kampala to other parts of Uganda.The study was embedded in a case control study that evaluated the association between malaria and overnight travel from Kampala [8].

## Methods

### Study design

A qualitative cross-sectional design was employed in August and September 2019. The study targeted patients with history of overnight travel out of Kampala within the previous 60 days. The purpose of the study was to analyse local perceptions of use of LLINs and subsequent malaria prevention practices during overnight travel. Overnight travel was defined as travel out of the city of Kampala, with travellers spending at least one night outside of the city, in the last 3 months prior to the interview date. Use of malaria prevention methods, including LLINs, application of mosquito repellents as sprays or skin creams [18], wearing protective clothes [19] such as long sleeves, hats, jackets and socks [20] and the use of topical herbal medicine was studied.

### Study setting

The study was carried out at Naguru General Hospital in Kampala. This public hospital serves the inhabitants of Kampala metropolitan area, in the eastern part of the city. According to a recent Malaria Indicator Survey, prevalence of malaria by microscopy in children under 5 years old was lowest in Kampala (0.2%) and highest in the Karamoja (rural) region (34.3%) [21].

### Participant selection

Patients presenting to the out-patient department laboratory for a malaria test during the day from Monday to Friday who did not require hospitalization were approached for participation in the study as previously described elsewhere [8]. Briefly, patients were enrolled in a case-control study as cases if they had a positive test for malaria, and those with a negative test as controls, until 162 cases and 405 controls were enrolled. A study nurse reviewed hospital laboratory records to identify patients who were residents of Kampala with a history of recent overnight travel. Informed consent was obtained from eligible participants before interviews were conducted.

### Research team and reflexivity

The team employed two research assistants, a nurse and laboratory technician who participated in the screening process and were stationed at Naguru hospital. They referred patients to the social scientist who independently participated in the consenting process. The participants were encouraged to freely express themselves and were reminded that their identity would be kept confidential.

### Sampling

A total of 20 participants were purposively selected because of their history of overnight travel. They were recruited following the principle of saturation until no new information was emerging [22, 23].

### Data collection

Detailed data were collected on places where participants slept during travel including hotels, funerals and other people’s homes, and reasons for travel. In-depth interviews were conducted in English and the local language (Luganda) as appropriate, using a question guide. The interviews were conducted at the clinic immediately after testing on the same day and they lasted approximately 30–45 min. The interviews collected data on participants’ local understanding of malaria prevention during travel including perceptions and attitudes towards use of LLINs, sources of information about malaria prevention, challenges to prevention, and prospects for use of LLINs for malaria prevention during overnight travel. Participants’ knowledge of LLIN use was explored by asking “What are the causes of malaria during or after overnight travel?” Participants were then asked, “Have you ever heard of any malaria prevention measures?” All participants had heard of measures to prevent malaria and were asked “From where did you hear about malaria prevention measures?” Participants were further asked “Would you be willing to use LLINs in particular during overnight travel?” and “What is your understanding about the importance of using LLINs during overnight travel?” Participants were finally asked “What is your attitude and perception to use of LLINs during overnight travel?” (Table 1). Interviews were audio-recorded, with each reviewed for completeness and accuracy. Those conducted in Luganda were translated to English and saved for further analysis.

**Table 1:** Thematic categories from in depth interviews for overnight travellers in 2019

Theme	Category
Theme1: Prospects to use of malaria prevention methods	
Motivation for use of malaria prevention methods	Knowledge about preventive methods Affordability Causes of malaria during travel Positive perception about use of malaria prevention methods Health benefit
Theme 2: Challenges to use of malaria prevention methods	
Individual barriers	Time of travel Reason for travel Time for preparation Negative perceptions about malaria prevention methods
Logistical factors	Limited space for use of malaria prevention methods High cost of malaria prevention methods
Socio-cultural factors	Use of traditional medicine Cultural beliefs and practices
Theme 3: Reasons for travel	Social: (weddings, funerals, overnight prayers), Family obligations: (school, holiday for children) Work: (work/ business trips)
Theme 4: Places where participants slept	Hotels, funerals, conference halls, personal upcountry homes, other people’s homes
Theme 5: Source of information about malaria prevention	Sensitization campaigns at health facilities, media (radio and TV)
Willingness to use malaria prevention methods during travel	Yes No

### Analysis

Interviews were transcribed verbatim. Those conducted in the local language were translated to English and entered into NVivo (QSR international 2020). A team-based approach of thematic analysis was carried out. Data and recurring ideas were coded, and codes were aggregated into themes. This process involved carrying out multiple readings of the field scripts to understand the data and subsequent coding of the data. The first and the last authors (social scientists) were involved in applying and confirming the application of codes across all transcripts and disagreements were resolved by cross checking the recorded data. Coded extracts of data were developed into themes presented as sub-topics in this paper.

### Ethical approvals

Ethical approval for the study was obtained from Mulago Hospital Research and Ethics Committee (MHREC# 1592), the London School of Hygiene and Tropical Medicine Ethics Committee (LSHTM Ref: 16625), and the Uganda National Council of Science and Technology (UNCST Ref: SS 5012). All study staff were trained on procedures of acquiring consent and maintaining confidentiality during the study. Informed consent was obtained from all study participants before interviews were conducted. To ensure confidentiality, all participant names and other identities were anonymized.

## Results

### Socio demographic characteristics of the study population

Five-hundred and sixty-seven participants enrolled in a case-control study were approached and 30 participants purposively selected for participation in this study. Selected participants were further screened and 20 were enrolled. Reasons for exclusion included travel for more than 90 days, age under 15 years, and inability to provide consent. Of the enrolled, 13 were male and 11 had attained tertiary level or postgraduate education with a good level of understanding of malaria prevention (Table 2). A total of 16 participants were employed.

**Table 2:** Baseline characteristics of study participants

Characteristics	Frequency [n = 20, (%)]
Age range	17–62 years
Sex	
Female	7 (35)
Male	13 (65)
Education level	
No education	0
Primary	2 (10)
Secondary	7 (35)
Tertiary /university	9 (45)
Postgraduate	3 (10)
Occupation status	
Student	4 (20)
Unemployed	0
Employed	16 (80)
Categories of employment	
Transport industry (drivers, driver assistants)	5 (31)
Business people	3 (19)
Security guards	4 (25)
Causal laborers	2/(13)
Health workers	2 (13)
Malaria status	
Positive	13 (65)
Negative	7 (35)

Of the enrolled, 13 were male and 11 had attained tertiary level or postgraduate education with a good level of understanding of malaria prevention (Table 2). A total of 16 participants were employed, with 5 involved in transport (truck drivers, taxi drivers and their assistants), and 4 were business people who travelled frequently. Thirteen of the participants tested positive for malaria. Of the participants, 13, primarily truck drivers, seemed to be aware of the threat of malaria attributed to non-use of LLINs during overnight travel.

### Reason and time of travel and use of malaria prevention measures

Several reasons for travel were highlighted, including family obligations, school, holiday for children, work or business obligations, and social gatherings such as overnight prayers or attending weddings or burials. Participants reported that they preferred to travel during the night because they worked during the day. When preparing for trips, malaria prevention was usually not prioritized, and packing a LLIN was a rare practice. Most of the reasons for travel required the participants to move abruptly, without adequate time to acquire malaria prevention methods. One of the study participants shared his travel experience:

*“… it is always very abrupt; getting a net and buying repellents are one of the last things you think about”* (28-year-old male, university student).

Several travellers observed that because of the short and abrupt nature of their trips they preferred to carry small bags during travel, thus:

*“Because if you are going to travel, the last thing you are going to think about is whether you are going to be bitten by mosquitoes. You are not prepared for the night. So most probably you don’t have a mosquito net and it’s inconveniencing to everyone even to start packing a net.”* (27-year-old female, cleaner).

Use of mosquito nets was at times not a viable option, with limited space for travellers to hang the nets during travel, and while repellents were convenient to carry during travel, they were considered costly.

### Knowledge about malaria personal prevention methods

Study participants’ knowledge about the cause of malaria and available prevention methods was key in influencing usage of prevention methods during travel. The majority of participants noted using of LLINs at home rather than during travel and this was tied to the ease of use. A few of the participants had heard of repellents or used them before. The level of knowledge about malaria personal prevention methods affected their use. One female respondent seemed aware of the malaria prevention measures that could be used during travel:

The first malaria prevention method is sleep under a mosquito net, using mosquito repellents like ‘odomos’, use of the sprays and prophylaxis. (50-year-old female, farmer).

A few of the particpants couldn’t differentiate between the spread of malaria and influenza commonly known as musujja in Luganda. One female respondent linked the cause of malaria to sneezing and sharing of clothes thus:

*“Now when you have travelled, and you sit near this person who is having malaria and for you you’re not having, if they sneeze, you can get malaria. Even if you share clothes, even mucus can go on the cloth, if you use that cloth, you don’t know that thing (mucus) may still be there and you get malaria*”(21 year old female business woman).

Participants knew the importance of using malaria prevention measures during overnight travel to avoid malaria, and the social and economic costs involved, including the cost of malaria prevention measures, loss of time when they fall sick, disruption of activities and businesses, and loss of income. One participant observed how the cost of malaria prevention during travel was considered cheaper than what it would cost in case of contracting malaria. This implied being absent from work and loss of income, which influenced behaviour change towards use of malaria prevention measures:

*“Some people are employees and are required at their work places daily. Therefore, they have to be prepared for whatever situation that comes during travel. Then for the others, it’s that the malaria prevention options are not as expensive as the cure. For some of them, the conditions of their jobs are tough; the day one misses work is when they cut their salary and you miss a travel allowance. Therefore, he or she must have malaria prevention options.”* (28-year-old male, researcher).

Some travel destinations were perceived to be of low risk, and in such areas malaria prevention was thought to be unnecessary.

*“I know Kabale is a very low transmission area. So, at the back of my mind I don’t have many worries of getting malaria from Kabale. Even when I came, I didn’t test. I didn’t feel sick. If I compare that to the first day I went to Tororo, I knew it was a highly endemic area and there I had to sleep in a net everyday much as when I was still in Mbarara and Kabale where I never used to.”* (28-year-old female University student).

### Perceptions regarding malaria personal prevention methods

While participants had heard about malaria prevention measures, in some instances inaccurate knowledge about them shaped their perceptions. While overnight travellers rarely used LLINs because of the negative perceptions they had about those in lodging places being unhygienic, they did not use repellents either. LLINs provided by the Ministry of Health were considered to be poor quality, with chemicals that made people sceptical about using them.

*“For the government given nets, they are poor quality like I said. They have that itchy chemical and they are also very hard. Then still the net was on the bed when I travelled but it didn’t look nice and I wouldn’t use it even if there were mosquitoes, I would probably cover my head overnight.”* (29-year-old female, business woman).

Despite not using LLINs, they did not use any other malaria prevention measures such as mosquito repellents, which were considered easy to use among travellers. The repellents were reported to have a bad smell and to be dangerous to the skin, and others were perceived to cause diarrhoea, nausea and vomiting, and hallucinations, and to be dangerous to human health in general, as one of the travellers observed:

*“I hear that they cause cancer (repellents), and we often fear the imported things. Even if you tell someone that use the repellents, they argue that they have some side effects.”* (29-year-old female, business woman).

A few participants reported they used odourless repellents often meant for children, because of the good scent which sometimes were not very effective for adults.

The local understanding was that use of medications before getting a disease, before travel in this case, can lead to adverse effects to the body.

*“Personally, I have not used it (chemoprophylaxis), but I hear that some people take fansidar before they go to the village. I had a friend who told me that she would take fansidar whenever she went to the village to avoid getting malaria. I told her to stop taking fansidar because it will spoil her liver.”* (52-year-old female, farmer).

There appeared to be gender differences in the use of LLINs during overnight travel. Female participants were very keen about the quality of LLINs and less likely to use those in lodges they considered unhygienic and of poor quality while male participants were more likely to use them even for a short duration of time if available.

### Sources of information about malaria prevention

Frequent travellers were more informed and likely to use LLINs compared to people who seldom travelled. The variation in knowledge and use of malaria prevention methods could be attributed to the various sources of malaria health education in Uganda. Information about malaria prevention was often obtained from several sources, and mainly from LLIN sensitization campaigns at health facilities and from media (radio and television). Others recalled what they had learnt in school:

*“Some of this information we get from schools like you can be taught something from school and then with time when such happens to you, now you start recalling. One, sleep under mosquito nets. Second, don’t over stay outside.”* (28-year-old male, researcher).

Nevertheless, some participants’ understanding of malaria prevention while travelling was still lacking.

### Challenges and opportunities of using malaria prevention methods

#### Affordability of malaria personal prevention methods

The cost of malaria prevention methods was considered to be a deterrent to their use during overnight travel. Many overnight travellers lacked the funds needed to pay for preventive methods on the market and could rarely buy a spare LLIN to use during overnight travel. Many of the participants were security guards who worked in different places in the night, with minimal earnings. One participant highlighted the cost challenges of obtaining malaria prevention methods:

*“Now like me I am a security guard, however, most of those things* (malaria prevention measures*) require money. You see, most of the people here are not all that financially well off and cannot afford some of those things.”* (33-year-old female, security guard).

Particularly repellents, sprays or creams and chemoprophylaxis medications were perceived as expensive and considered to be for the rich class.

#### Logistical factors

The reasons for travel and logistical challenges played a key role in determining the use and non-use of malaria personal prevention methods. Limited space when sleeping outside the home and sleeping overnight in trucks for truck drivers and their assistants were some of the most common. Use of LLINs in the trucks was not a viable option, and while repellents were ideal, they were reported to be costly. One of the truck drivers who often slept in the truck shared his experience:

*“Our condition is hard, we sleep in the trucks which are filled with goods that you are transporting. There is a small space for one person to sleep, which I share with my assistant, so you cannot even consider hanging a bed net.”* (62-year-old male, truck driver).

Most travellers reported that when they travelled they had no control over sleeping conditions. A participant who attended overnight prayers and had no control over the sleeping conditions at a conference explained:

*“I went to Rukungiri, and I slept, in fact we reached there around 11pm and slept in an open place (*conference room*) without any mosquito net.”* (33-year-old female, security guard).

One participant noted that in a bid to overcome the logistical challenges with use of LLINs during travel, consideration could be made to use of pop-up nets and repellents which were considered very easy to use during travel:

*“I think there are nets that you can easily set up (*pop-up*). While travelling, a repellent would be the easiest thing to use because even if you have a net that you can set up, you might not have enough space to do that.”* (28-year-old female, university student).

However, due to limited knowledge of pop-up nets, and the unaffordable cost of repellents, these two malaria prevention methods were rarely used by the travellers.

#### Cultural beliefs and practices

Depending on the reason for the overnight travel, the travellers were expected to conform to the behavioural norms of local communities. Failure to behave as expected was considered disrespectful and largely unacceptable. For instance, in most cultures in Uganda it would be perceived as a social deviance to use a mosquito net during funeral rites. Traditionally people are expected to sit around a fire and console with the bereaved:

*“We don’t sleep under the net when its burial time… You cannot decide to put your net, who are you? How important are you? How arrogant are you? So, most of us in Teso, we don’t even sleep when at a funeral. We sit out around the fire or even within a house, and in big numbers, so one cannot use a mosquito net.” *(52-year-old female, farmer).

In such circumstances, very few travellers slept under LLINs. Others reported wearing long-sleeved clothing and hats during overnight travel and related activities, including attending parties, funerals, or other business and social engagements.

Lastly, several travellers reported use of herbal medicines to prevent malaria before travelling to villages. *Aloe vera* (locally known as Kigajji and kazire) and *vernonia amygdalina* (locally known as mululuza), were commonly used both before or during travel. The herbal medicines were usually sold to travellers in buses as they travelled to their destinations. These were usually in form of dry roots and/or leaves processed in powder form, and sometimes cooked or squeezed, and mixed in water for drinking and bathing.

## Discussion

Within sub-Saharan Africa, travellers from areas of lower to higher transmission intensity are potentially at high risk of malaria infection and yet use of LLINs during travel is limited [24]. In this urban population, perceptions, attitudes and experiences were explored regarding use of LLINs for malaria prevention by Kampala residents travelling to other parts of Uganda. Use of malaria prevention methods during overnight travel was influenced by social, economic and logistical reasons, and cultural beliefs and practices. The key findings were that non-use of malaria prevention measures was heightened during travel because of: (1) the impression that LLINs in lodging places were unhygienic; (2) cultural beliefs about use of LLINs during social functions; and, (3) challenges in planning ahead of travel. The abrupt nature of most of the trips made it hard for the travellers to prioritize malaria prevention methods when planning for a trip away from home.

Participants reported that they preferred to travel during the night because they worked during the day. The most common reasons for travel were family obligations, work or business obligations, or social gatherings such as weddings or burials that compelled one to spend nights out of home when use of LLINs would be inappropriate. It has been documented in other studies that potential exposure to malaria is influenced by the duration of travel, purpose of travel and participation in late outdoor activities [1]. Travel plays a crucial role in the spread of malaria when individuals travel to areas with greater transmission risk than at their homes [1, 14, 25, 26]. The implication is that although a number of malaria prevention methods exist, most of them were developed for fixed settings [27]. While use of LLINs were perceived to be effective malaria protective measures [28], their use was limited in situations for overnight travellers and largely meant for malaria prevention in the home [27]. Use of LLINs outside of home is very challenging with limited space for a traveller to hang a net. This can be addressed by increased sensitization messages about the use of LLINs particularly those appropriate for travel, such as the pop-ups, and adoption of use of repellents during social events. However, the popup nets are currently available only for babies in Uganda. They would be an innovative option if treated and considered for adult travellers in Uganda.

Negative perception about the quality and hygiene of LLINs was considered key in use of malaria prevention during overnight travel. Good quality, smell and material of LLINs are influential in use of malaria prevention methods during overnight travel, consistent with findings of similar studies [29–32]. Gender differences were observed in the use of LLINs in lodging places, with females less likely to use those considered of poor quality and unhygienic, compared to males who seemed not to mind. This is consistent with a study conducted in Uganda that showed that women were less likely than men to sleep under LLINs during travel [24]. The negative perception and cultural beliefs community members held about people who used LLINs, especially during social events, hindered their use. Social approval [33] was a key factor in use of malaria prevention methods. People are influenced by peer pressure and will not use malaria prevention methods if not socially approved. People who used LLINs during social events such as funerals were considered proud, and it was an act not culturally accepted by community members. Despite the negative perception held by some of the particpants about the unpleasantness of repellents as a danger to skin and cause of cancer, they were highly recommended to travellers. Repellents are portable thus easy for the travellers to carry. Similar studies have shown their safety and effectiveness in reducing the risk of infection by malaria [34–36].

Non-use of LLINs can partly be explained by reason for travel, some of which are logistical barriers [25, 27]. The majority of the travellers moved in the night and used both private and public means, which made it impossible to use LLINs. The use of LLINs in both situations was impossible because of the limited space to hang the LLINs. However, those who used public transport were more at risk of being bitten by mosquitoes as they waited for means of transport outside at night. This is linked to the biting tendencies of the female anophelese mosquitoe from dusk to dawn [37–39].

Occasionally they preferred to carry small bags, which could not accommodate LLINs, which are not portable and convenient during travel. This can be addressed by encouraging travellers to utilize other malaria prevention measures such as mosquito repellents. The Ministry of Health should increase sensitization messages about the use of LLINs among travellers regardless of the perception of LLINs being too bulky to carry during travel.

The study had several limitations. First, findings are not generalisable to the entire Ugandan population but include important information that may inform policy and contribute to malaria prevention and control education programming in Uganda and other similar settings. Second, the findings were mainly focused on the use of LLINs and not on all personal malaria prevention methods. However, this information can also apply to non-use of other malaria prevention methods while travelling and can help improve usage. Third, the information collected is highly subjective, but can benefit from the principle of transferability of knowledge through systematic comparisons and sharing of lessons learned. Moreover, purposive sampling was used to ensure that views that were lived experiences of overnight travellers were captured.

## Conclusions

Despite travellers’ knowledge of malaria prevention measures, LLIN use was limited. The importance of malaria prevention among travellers from low to high transmission settings needs to be prioritized, even in endemic countries where there is variation in transmission. Truck drivers should be sensitized to use the available space at the back of the trucks for hanging nets and using pop-up nets. This calls for increased behaviour change oriented communication to improve on traveller preparedness in terms of malaria prevention methods in the promotion of use of repellents in situations where LLINs may not be feasible. Examining the local understanding of malaria prevention methods in urban and local settings is important. Malaria Control Division should use educational messages to improve awareness of the risks of getting malaria during overnight travel which will help better protect overnight travellers.

## Supplementary Information

**Additional file 1.** The in-depth interview guide.

## Data Availability

The in-depth interview guide is attached as an Additional file 1. Source data are in the form of a data base and recorded voice which could break anonymity. All IDIs have been anonymized, transcribed, and translated into English. Due to the sensitivity attached to this material it will not be available online. However, these anonymized data may be available on reasonable request from the author.
