# Supplementary material for: Challenges and opportunities for use of long-lasting insecticidal nets to prevent malaria during overnight travel in Uganda: a qualitative study
Source: Malar J. 2021 Jun 26;20:283. doi: 10.1186/s12936-021-03811-1 (PMC8235645; doi:10.1186/s12936-021-03811-1)
Supplement: Supplementary file 1 — Additional file 1. The in-depth interview guide. [file 12936_2021_3811_MOESM1_ESM.docx]

**Factors associated with the risk of malaria during recent overnight travel out of Kampala city: In Depth Interview guide**

| Demographics | | |
| --- | --- | --- |
| Study ID [____\|____] **Date** [____\|____] / [­____\|____] / ­­[____\|____] | | |
| day month year | | |
| **Gender** 1 = Male 2 = Female [____] **Age in years** [____\|____] | | |
| Highest level of education or qualification achieved | | |
| 1 = Primary (P1 — P7)  2 = Secondary UCE (S1 — S4)  3 = High School Education UACE (S1 – S6)  4 = Certificate | 5 = Diploma  6 = Bachelor’s degree  7 = Master’s degree  8 = PhD | [____] |
| **Introduction** | | |
| *“Hello, my name is ……….. I am interested in discussing with you a few questions about your experiences, opinions about risk of malaria during a recent trip that you made out of Kampala and spent a night away. A note-taker will be writing down what you say for our record and we will record the interview using a digital recorder; these notes will be kept secure and your name will not be used anywhere. Your answers will be looked at together with responses from other respondents and you will not be identifiable in any reports.*  *Now we request that we all switch off our mobile phones so that we are not distracted.* | | |
| **Topics and probes** | | |
| **MALARIA**   1. Tell me what you know about how malaria is spread? (Probe if ever suffered from malaria, feeling about suffering from malaria, how did participants tell they had malaria, what care do they do they receive for malaria? 2. How does community perceive people with malaria (probe for effects of malaria to an individual, family and community)? 3. Can you kindly tell me about what kind of things people do when they get malaria (probe for use of herbs, attending facility, buy drug from a drug shop/pharmacy) 4. Tell me what you know about Malaria prevention options? (Probe for bed net, mosquito repellants, mosquito sprays, use of medication, herbal use etc.) 5. Where do you obtain the malaria prevention methods? (Probe for motivation to use these methods, probe for perception of risks and benefits)? 6. Can you share any challenges that people face in accessing Malaria prevention options?    1. Probe for cost - facility related, availability    2. Probe for difficulties/ barriers    3. What makes it easy, and suggestions on improvements to make 7. Can you tell me what factors you take into consideration when making the decision to use malaria control measures? (Probe for cost)  - If risk is a factor, probe for the likely risks that can affect use of malaria control strategies (side effects), can you share with us some of your fears? | | |
| **TRAVEL AND MALARIA**   1. Can you kindly share your experience during your last overnight travel (probe for reasons for the travel, time of travel, places travelled to and how often they travel) 2. In your opinion, how does overnight travel affect use of malaria control strategies (probe for where to hang the net, going to the discos, hanging out in the bar) 3. Malaria control interventions are of importance to us. What is your opinion about this? 4. What are some of the reasons that would motivate you to use malaria prevention measures?   b) What are some of the challenges to use of malaria prevention measures?   1. What are the solutions to use of malaria prevention measures among overnight travelers? 2. Is there anything else you want to tell me about Malaria? | | |
